# Oral Bacterial Infection and Shedding in *Drosophila melanogaster*

**DOI:** 10.3791/57676

**Published:** 2018-05-31

**Authors:** Jonathon A. Siva-Jothy, Arun Prakash, Radhakrishnan B. Vasanthakrishnan, Katy M. Monteith, Pedro F. Vale

**Affiliations:** ^1^Institute of Evolutionary Biology, School of Biological Sciences, University of Edinburgh; ^2^IGDR - CNRS UMR 6290; ^3^Centre for Immunity, Infection and Evolution, University of Edinburgh

**Keywords:** Biology, Issue 135, Infection, immunity, *Drosophila*, oral infection, bacterial shedding, Relish, Dcy

## Abstract

The fruit fly *Drosophila melanogaster* is one of the best developed model systems of infection and innate immunity. While most work has focused on systemic infections, there has been a recent increase of interest in the mechanisms of gut immunocompetence to pathogens, which require methods to orally infect flies. Here we present a protocol to orally expose individual flies to an opportunistic bacterial pathogen (*Pseudomonas aeruginosa*) and a natural bacterial pathogen of *D. melanogaster* (*Pseudomonas entomophila*). The goal of this protocol is to provide a robust method to expose male and female flies to these pathogens. We provide representative results showing survival phenotypes, microbe loads, and bacterial shedding, which is relevant for the study of heterogeneity in pathogen transmission. Finally, we confirm that *Dcy* mutants (lacking the protective peritrophic matrix in the gut epithelium) and Relish mutants (lacking a functional immune deficiency (IMD) pathway), show increased susceptibility to bacterial oral infection. This protocol, therefore, describes a robust method to infect flies using the oral route of infection, which can be extended to the study of a variety genetic and environmental sources of variation in gut infection outcomes and bacterial transmission.

**Figure:**
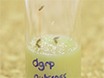


## Introduction

The fruit fly (also known as the vinegar fly), *D. melanogaster*, has been extensively used as a model organism for infection and immunity against a variety of pathogens^1^,^2^. This work has offered fundamental insights into the physiological consequences of infection and was also pioneering in unraveling the molecular pathways underlying the host immune response against parasitoid, bacterial, fungal, and viral infections. This knowledge is not only useful to understand the innate immune response of insects and other invertebrates, but because many of the immune mechanisms are evolutionarily conserved between insects and mammals, *Drosophila* has also spurred the discovery of major immune mechanisms in mammals, including humans^3^.

Most work on *Drosophila* infection and immunity has focused on systemic infections, using inoculation methods that deliver pathogens directly into the body of the insect by pricking or injection^4^,^5^,^6^. The advantage of these methods in allowing the delivery of a controlled infectious dose is clear and supported by a large body of work on systemic infections. However, many naturally occurring bacterial pathogens of *D. melanogaster* are acquired through feeding on decomposing organic matter where gut immunocompetence plays a significant role in host defence^7^,^8^,^9^,^10^,^11^,^12^,^13^,^14^,^15^. Experiments that employ systemic infections bypass these defenses, and, therefore, provide an altogether different picture of how insects mount defenses against natural pathogens. This is especially relevant if the aim of the work is to test predictions about the ecology and evolution of infection, where the use of natural pathogens and routes of infection is important^16^,^17^. Recent work has highlighted how the route taken by pathogens significantly affects disease outcome^18^,^19^, elicits distinct immune pathways^20^,^21^, can determine the protective effect of inherited endosymbionts^16^, and may even play an important role in the evolution of host defenses^17^.

Another reason to employ oral routes of infection is that it allows the investigation of the variation in pathogen transmission by measuring bacterial shedding during fecal excretion following oral infection^22^,^23^,^24^. Understanding the sources of host heterogeneity in disease transmission is challenging in natural populations^25^,^26^, but measuring components of transmission, such as pathogen shedding, under controlled laboratory conditions offers a useful alternative approach^27^. By feeding flies bacteria and measuring bacterial shedding under a variety of genetic and environmental contexts in controlled experimental conditions, it is possible to identify sources of variation in transmission among hosts.

Here, we describe a protocol for orally infecting *D. melanogaster* with bacterial pathogens, and for quantifying the bacterial growth and shedding that follows (Figure 1). We describe this protocol on two *Pseudomonas *bacteria: a virulent strain of the opportunistic pathogen *P. aeruginosa* (PA14), and a less virulent strain of the natural fly pathogen *P. entomophila*. Pseudomonads are common gram-negative bacteria with a broad host range, infecting insects, nematodes, plants, and vertebrates, and are found in most environments^4^,^6^. Enteric infection of *Drosophila* by *P. aeruginosa* and *P. entomophila* results in pathology to intestinal epithelia^12^,^13^,^14^,^15^,^28^. While we focus on these two bacterial pathogens, the methods described here can in principle be applied to any bacterial pathogen of interest with minor modifications. Following oral exposure, we measure post-infection survival, and measure the microbe load within individual flies and the viable microbes shed into the environment, expressed in colony forming units (CFUs). Finally, because gut immunocompetence results from a combination of epithelial barrier and humoral responses, we also measure the survival of fly lines where these defenses are disrupted. Specifically, Drosocrystallin (*Dcy)* mutants have been previously shown to be more susceptible to oral bacterial infection due to a depleted peritrophic matrix in the gut^29^. We also measure survival in a Relish (*Rel*) mutant which is impeded from producing antimicrobial peptides against Gram-negative bacteria via the IMD pathway^30^.

## Protocol

### 1. Maintain Flies

1. Maintain flies in 23 mL plastic vials containing 7 mL of freshly made Lewis medium (modified from reference^31^; 1 L triple distilled H_2_O, 6.1 g agar, 93.6 g brown sugar, 68 g maize, 18.7 g instant yeast, 15 mL Tegosept anti-fungal agent) in incubators at 25 ± 1 °C, in a 12 h:12 h light:dark cycle with ~60% humidity. Plug the vials with non-absorbent cotton wool.
2. After every 14 days, transfer 20–30 adults to a new food vial, with instant, dry yeast added to the surface, for 2–3 days to allow egg-laying to occur. After this time period, ensure that the eggs are visible on the surface of the food. Remove adult flies. NOTE: This keeps flies in the vials as single generation, age-matched populations.
3. Leave the eggs to develop. NOTE: At 25 °C, adult flies start to eclose from pupae on day 11 and continue over days 12–14.

### 2. Prepare Experimental Flies

1. Collect the eggs of the parent generation in a population/embryo collection cage on a 75 mL apple-agar plate (1 L triple distilled H_2_O, 30 g agar, 33 g sucrose, 330 mL apple juice, 7 mL Tegosept anti-fungal agent) with a yeast paste spread (mix dry yeast with water to a peanut butter-like consistency). Add water-soaked cotton wool to the cage to provide moisture. NOTE: To avoid confounding effects caused by differences in larval rearing density, it is important that experimental flies in different vials are reared in similar densities. The above step is performed to avoid confounding effects.
2. Incubate for 24 h at 25 °C in a 12 h:12 h light:dark cycle until egg-laying has occurred. If there are too few eggs after 24 h, provide a longer habituation period. Replace apple-agar plates and allow egg-laying to occur for a further 24 h.
3. Take egg-laden apple-agar plates from the population cage. Remove the remaining yeast paste and any dead flies from the agar’s surface.
4. Submerge the agar in 20 mL of 1x phosphate-buffered saline (PBS) and gently dislodge the eggs from the apple-agar with a fine paintbrush. While suspended in PBS, transfer the eggs to a 50 mL centrifuge tube and leave for 5 min so the eggs sink to the bottom. NOTE: Most eggs are found on the outer edge of the agar.
5. Remove by cutting the bottom 4 mm of a p1000 filtered pipette tip and use the pipette tip to draw 1 mL of solution, taken from the bottom of the 50 mL centrifuge tube. Transfer this to a 1.5 mL microcentrifuge tube and allow it to settle. NOTE: When pipetting up eggs, snap-releasing the plunger is more efficient than a gentle release.
6. Remove by cutting the bottom 4 mm of a p20 filtered pipette tip. Set the pipette to a desired volume and draw from the bottom of the microcentrifuge tube. NOTE: With practice, a volume of 5 µL contains roughly 100 eggs.
7. Dispense the collected eggs onto the food and leave them to develop for the required amount of time.

### 3. Bacterial Culture

1. To grow *P. entomophila* and *P. aeruginosa *cultures, inoculate 10 mL of Luria-Bertani (LB) broth with 100 µL of a frozen bacterial stock at 30 °C (*P. entomophila)* and 37 °C (*P. aeruginosa*), respectively. Shake at 150 rpm overnight. Ensure that the bacterial culture reaches the saturation phase.
2. To ensure the bacteria used for inoculating the flies are in the exponential phase and rapidly replicating, inoculate the overnight culture into a new subculture, of a desired volume, the following morning. Ensure that the pre-inoculum is 10% of the total volume of the subculture culture. NOTE: Oral infection requires high . It is therefore necessary to grow a substantial volume of bacterial culture so that enough inoculation culture can be produced for the desired dose and experimental size. Calculate how much subculture is needed to produce the required infectious doses using the equation M_s_V_s_ = M_i_V_i_, where M represents a culture’s optical density measured at 600 nm (OD_600_) value and V represents its volume. Subscript letters refer to whether the culture is used as a subculture (s) or an infectious dose (i).
3. Grow this subculture in a 2 L conical flask in a volume such that the subculture’s surface falls (at most) just above the beginning of the flask’s slope. Do not fill above this mark as it will stunt the growth of bacteria.
4. Ensure the bacteria in this subculture are in the exponential growth phase by measuring the OD every 30 min. NOTE: This occurs after 3–5 h, where the subculture reaches an OD_600_ between 0.6–0.8.
5. Pour equal volumes of this subculture across 50 mL centrifuge tubes and spin the subculture at 2,500 x g for 15 min at 4 °C to pellet the bacteria. Once pelleted, remove and then spin the supernatant again at the above conditions to confirm the removal of the vast majority of bacteria. NOTE: A pellet of negligible size (smaller than 1 mm in height) confirms this.
6. Combine the bacterial pellets of the separate tubes by re-suspending them in 5 mL of subculture supernatant and recombining these solutions in a single 50 mL tube. Spin this concentrated culture at 2,500 x g for 15 min at 4 °C to pellet the bacteria.
7. Remove the supernatant and re-suspend the final bacteria pellet in 5% sucrose water solution. Check the OD and adjust to the desired infectious dose (OD_600_ = 100 for *P. entomophila*^8^ and OD_600_ = 25 for *P. aeruginosa*^16^,^28^), by re-suspending the pellets in 5% sucrose water solution to the required volume. NOTE: The amount of 5% sucrose water solution to be added can be calculated using the equation in step 3.2.1 (M_s_V_s_ = M_i_V_i_).

### 4. Orally Infecting Flies

1. To ensure oral infection, starve the flies for 2–4 h before exposure to bacteria by transferring the flies to standard agar vials (1 L triple distilled H_2_O, 20 g agar, 84 g brown sugar, 7 mL Tegosept anti-fungal agent).
2. Prepare infection vials while flies are being starved. Make a *Pseudomonas* infection vial by pipetting 500 µL of standard sugar agar into the lid of a 7 mL sample tube and leave it to dry. Place a disc of filter paper in the lid and pipette 100 µL of bacterial culture directly onto the filter disc. For control infections, replace the bacterial culture with the same volume of 5% sucrose water solution on the filter paper.
3. Add single flies to the sample tube and leave for 18–24 h.
4. To confirm oral infection, first surface-sterilize the flies immediately after bacterial exposure, by placing them in 100 µL of 70% ethanol for 20–30 s. Remove the ethanol and add 100 µL of triple distilled water for 20–30 s before removing the water. Add 100 µL of 1x PBS and homogenize the fly.
5. Transfer the homogenate to the top row of a 96-well plate and add 90 µL of 1x PBS to every well below.
6. Serially dilute this sample to distinguish a range of CFU values. Take 10 µL of the homogenate in the top well and add this to the well below. Repeat this step with the second well, transferring 10 µL to the third well, and so on, for as many serial dilutions as required. NOTE: It important that new pipette tips are used for each set of dilutions.
7. Plate the serial dilutions on an LB nutrient agar plate in 5 µL droplets, to ensure all droplets remain discrete.
8. Incubate the LB Agar plates overnight at 30 °C and 37 °C for *P. entomophila *and *P. aeruginosa*, respectively and count visible CFUs. NOTE: While *Drosophila *gut microbes require distinct anaerobic growth conditions, selective medium, for example *Pseudomonas* Isolation Medium (PIM), may be used to make sure only *Pseudomonas* CFUs are counted.
9. Calculate the number of CFUs per fly by counting the number of colonies present at the serial dilution where 10–60 CFUs are clearly visible. Then multiply by the dilution factor present to calculate the number of bacteria per fly.
10. Perform statistical analysis. Where necessary, transform the CFUs per fly to a normal distribution. Do this by log-transformation. Once transformed, use Generalized Linear Models (GLMs)^30^,^31^,^32^ to test how treatment groups differ in CFUs per fly (using commonly available statistical software packages such as R^33^). NOTE: The remaining fly homogenate can be used for measuring gene expression through quantitative reverse transcription PCR (RT-qPCR) analysis. Fix the homogenate in 50 µL of RNA isolation reagent, extract RNA, and quantify specific immune gene titers by RT-qPCR (see *e.g.*, Gupta and Vale^16^ for a detailed protocol). The expression of specific immune gene transcripts should be normalized to the transcript levels of a housekeeping gene (*i.e.*, rp49) and expressed as a fold change relative to control flies using the 2^−ΔΔCt^ method^31^,^32^,^33^,^34^.

### 5. Recording Survivorship Following Infection

1. Infect flies orally as described in step 4.2.
2. Transfer the infected or control flies from their respective infection vials into standard Lewis vials and keep in an incubator at 25 °C in a 12 h:12 h light-dark cycle (or desired conditions). Keep flies until they are dead.
3. Count the number of living or dead flies in each vial every day, or as often as required.
4. Transfer the flies to new vials every 5 days to avoid the flies getting stuck in the food.
5. Present these data as Kaplan-Meier (KM) survival curves or Mean ± SE proportional survival plots. To analyze the effect of several factors and/or their respective interactions with one another use a statistical package (for example, the package “survival” in R^33^) to run a survival analysis such as the Cox Proportional Hazards model^35^.

### 6. Measuring Bacterial Load

1. At the desired time point, transfer a single infected fly to a sterile 1.5 mL microcentrifuge tube.
2. Surface sterilize the flies as described in step 4.4.
3. Homogenize the fly and quantify the bacterial load using the protocol described in steps 4.5–4.10.

### 7. Measure Bacterial Shedding

1. Measure the shedding alongside the internal load.
2. After infection, transfer single flies to 1.5 mL microcentrifuge tubes containing ~50 µL of Lewis medium for 24 h.
3. Remove the flies for the internal load measurement (see step 6) and wash the tubes with 100 µL of 1x PBS by vortexing heavily for 3 s.
4. Measure the CFUs in this wash by plating on LB nutrient agar using the same protocol as described in steps 4.6–4.8.
5. After infection, transfer single flies to 1.5 mL microcentrifuge tubes containing ~50 µL of Lewis medium for 24 h.
6. Transfer the flies to new microcentrifuge tubes containing ~50 µL of Lewis medium for a further 24 h. Wash the contaminated tubes with 100 µL of 1x PBS by vortexing heavily for 3 s.
7. Measure the CFUs in this wash by plating on LB nutrient agar using the same protocol described in steps 4.6–4.8.
8. Repeat steps 7.2 and 7.3 and record fly mortality at every transfer.

## Representative Results

Here, we present illustrative results from experiments where *D. melanogaster *was orally infected with *P. aeruginosa *or *P. entomophila*. Figure 2 demonstrates the successful oral infection of flies following a 12 h or 24 h exposure period to bacterial cultures of OD_600_ = 25 and 100 for *P. aeruginosa *(Figure 2A) and *P. entomophila *(Figure 2B**, C**), respectively. Figure 2B illustrates the importance of using a more concentrated culture of *P. entomophila, *shown by the increase in bacterial load when flies are exposed to bacterial cultures of greater optical density. Male and female Oregon R (OreR) flies clear *P. aeruginosa* infection at the same rate (Figure 3) and shed the same number of *P. aeruginosa* CFUs (Figure 4A). When infected with *P. entomophila *however, male and female OreR flies differ in the number of bacteria shed, in a manner that changes over time (Figure 4B). Males and females die from *P. aeruginosa *(Figure 5A) and* P. entomophila *(Figure 5B) at different rates. We also see that *Dcy* mutants (which lack the protective peritrophic matrix in the gut epithelium) and Relish mutants (which lack a functional IMD immune pathway), show decreased survival following *P. entomophila *and *P. aeruginosa *oral infection (Figure 5C).

**Figure:**
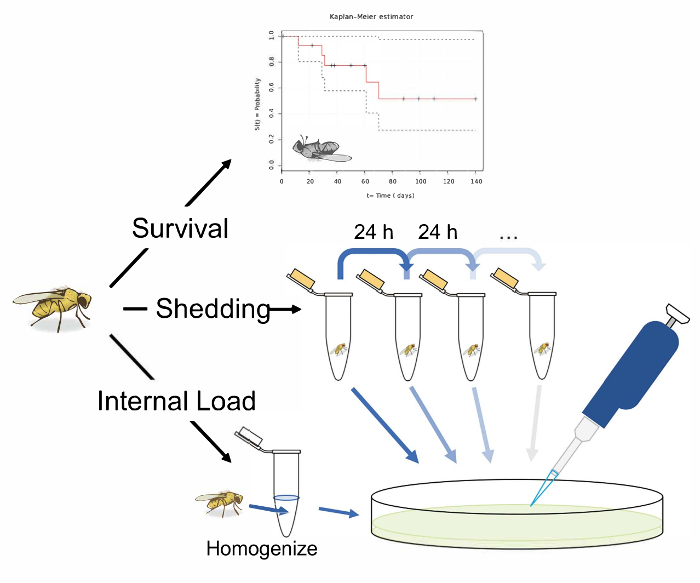

**Figure 1: Schematic overview of protocols for measuring survival, shedding, and internal bacterial load following oral infection in *Drosophila melanogaster*.** An illustration of 3 potential experiments following the oral infection of *D. melanogaster*. Measure the 'survival' by transferring single flies to vials and recording their infected lifespan. Measure 'shedding' by transferring single flies to 1.5 mL microcentrifuge tubes with 50 µL of Lewis medium in the cap. After 24 h in the tube, remove the fly and vortex the tube with 100 µL of 1x PBS. Remove and plate this solution on LB nutrient agar to calculate the bacterial shedding. Measure the shedding in the same fly longitudinally, by transferring flies to fresh tubes with Lewis medium in the cap after 24 h, and washing and plating the now contaminated tube. A fly's 'internal load' can be measured by taking an infected fly, surface sterilizing it, and homogenizing it before finally plating the homogenate on LB nutrient agar. This can be performed after shedding has been measured to calculate how the 'internal load' and shedding correlate. The fly illustration used in this figure was originally drawn by B. Nuhanen^36^. The authors have modified it to accompany the example Kaplan-Meier curve which is taken from Wikimedia Commons^37^. All other illustrations are original. Please click here to view a larger version of this figure.

**Figure:**
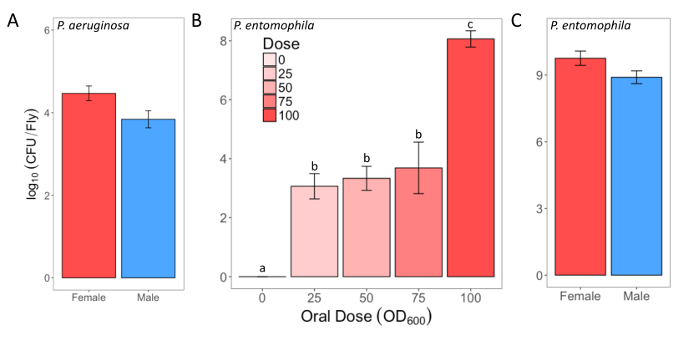

**Figure 2: Infectious dose of bacteria following oral infection. (A) **Infectious dose of male and female Oregon-R flies following exposure to a *P. aeruginosa *culture (OD_600_ = 25) for 12 h. The mean and SE were calculated from 3 males and 3 females. **(B) **Infectious dose of outcrossed wild-type females following exposure to one of four *P. entomophila *cultures (OD_600_ = 100, 75, 50, and 25) or control 5% sucrose solution for 24 h. The statistical difference of (F_3,76_ = 18.567, *p* <0.001) in the infectious dose between exposure treatments is denoted by differing letters above bars. The means were calculated from 5 flies for the OD_600_ = 0 dose, and 18-20 for all other doses. **(C) **The infectious dose of male and female Oregon-R flies following exposure to *P. entomophila *culture (OD_600_ = 100) for 24 h. The mean and SE were calculated from 20 males and 20 females. Please click here to view a larger version of this figure.

**Figure:**
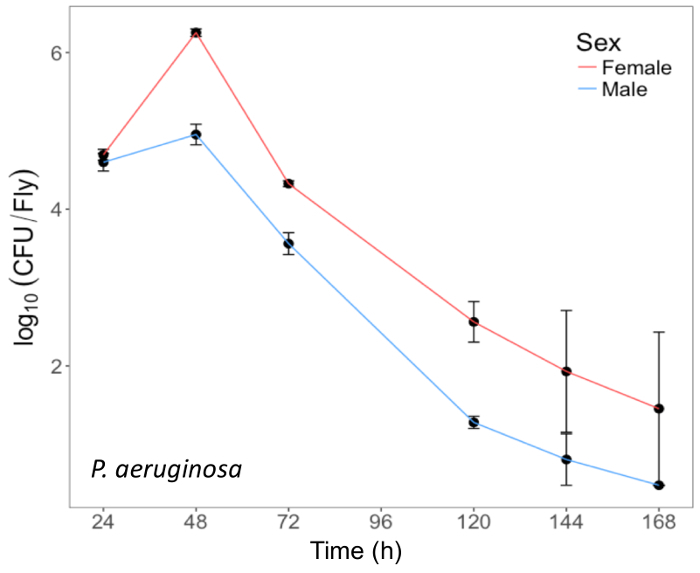

**Figure 3: Internal *P. aeruginosa *load in flies after oral infection. **Mean ± SE bacterial load of male and female Oregon-R flies following oral infection with *P. aeruginosa *(OD_600_ = 25) up to 168 h post-infection. The mean and SE of each time point are calculated from 3 individuals. A fly's internal bacterial load significantly changes over time (*p* <0.001). Please click here to view a larger version of this figure.

**Figure:**
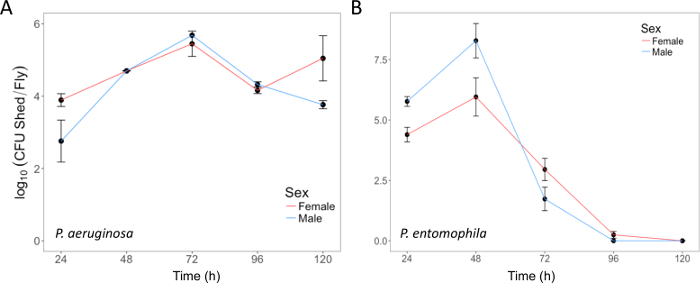

**Figure 4: Bacterial shedding following oral infection. (A)***P. aeruginosa *shed by the same flies used in Figure 3, up to 120 h post-infection. The mean and SE were calculated from 3 males and 3 females.** (B) ***P. entomophila *shed by male and female Oregon-R flies following oral infection with *P. entomophila *(OD_600_ = 100) up to 120 h post-infection. The mean and SE were calculated from 34 males and 38 females. For both* P. aeruginosa *and *P. entomophila*, the number of CFUs shed by a fly significantly changes over time (*p* <0.001). Please click here to view a larger version of this figure.

**Figure:**
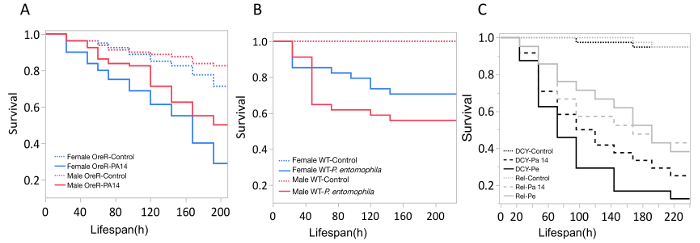

**Figure 5: Survival of flies following bacterial oral infection.** Kaplan-Meier (KM) survival curves of **(A) **Oregon-R male and female flies following oral infection with *P. aeruginosa *(OD_600_ = 25) or control 5% sucrose solution. The KM survival curve was calculated from 4 vials of 20 flies per treatment group. **(B)** OreR male and female flies after oral infection with *P. entomophila *(OD_600_ = 100). The KM survival curve was calculated from 4 single control flies and 34 infected flies for both males and females. **(C) **Immune mutants: *Dcy *(Drosocrystallin-peritrophic matrix mutant) and *Rel* (Relish-IMD mutant), exposed to *P. entomophila* (Pe), *P. aeruginosa* (Pa14), or a control 5% sucrose solution. All infected groups die significantly faster than the control flies (*p* <0.001). Please click here to view a larger version of this figure.

## Discussion

We present a protocol for reliably orally infecting *D. melanogaster *with bacterial pathogens. We focus on *P. aeruginosa *and *P. entomophila*, but this protocol can easily be adapted to enable infection of other bacterial species, *e.g.*, *Serratia marcescens*^7^*. *Key aspects of this protocol will vary between bacterial species. Accordingly, the most efficient infectious dose, corresponding virulence, and host genotype susceptibility should all be considered and ideally tested in pilot studies. Exposing flies to bacterial cultures of a range of optical densities and measuring their infectious dose and survival is an appropriate starting point when working with new bacterial species or fly lines.

Protocol steps such as fly starvation prior to feeding and re-suspending bacterial pellets in 5% sucrose solution are commonplace in oral infection and increase the reliability of bacterial infection during exposure^7^,^8^,^9^,^10^. However, it is important to note that during exposure, flies essentially live on a surface of bacterial culture. In the process of walking on this culture, bacteria will become lodged on the fly's surface, especially on the cuticle or around the bristles^24^. These epicuticular bacteria, do not reflect a successful enteric infection but would still be detected by the fly homogenization and plating. To reduce the potential for false positives, it is essential to surface sterilize flies through immersion in 70% ethanol for up to 1 min.

When considering bacterial shedding rates, oral infection is essential. The number of pathogens a host releases into the environment is often difficult to measure and the internal load is often taken as a proxy for the severity of infection and therefore transmission^26^,^27^. Measuring bacterial load alongside bacterial shedding allows an examination of the relationship between these two important components of disease severity and spread^38^. One limitation of the method presented is that assaying the internal bacterial load of flies requires destructive sampling. This makes it difficult to investigate longitudinal trends of pathogen growth and clearance within the same individual. However, it is possible to overcome this limitation by destructively sampling cohorts of individuals at different stages of infection, under the assumption that the average microbe load in each cohort reflects the longitudinal pathogen dynamics within any given individual. Bacterial shedding does not suffer from the same limitations, and we offer examples of how shedding can be quantified in a cross-sectional sample, or longitudinally to investigate how shedding changes within an individual over time.

Many host and pathogen traits jointly determine an individual's propensity to transmit disease^25^,^26^,^39^. While the significance of these traits likely varies between host-pathogen systems, shedding is likely a major determinant of fecal-oral transmission. The ability to measure bacterial shedding opens the opportunity to test this assumption. Having characterized host-pathogen dynamics in a desired panel of fly lines, experimenters could orally infect individuals, and place them alongside uninfected, susceptible hosts during their infectious periods. These 'recipient' flies could then be assayed for internal bacterial load at various time points as a way of directly measuring transmission.
